# Combined interaction of fungicides binary mixtures: experimental study and machine learning-driven QSAR modeling

**DOI:** 10.1038/s41598-024-63708-2

**Published:** 2024-06-03

**Authors:** Mohsen Abbod, Ahmad Mohammad

**Affiliations:** Department of Plant Protection, Faculty of Agriculture, Al-Baath University, Homs, Syria

**Keywords:** SVM, ANN, Synergism, Antagonist, Pesticide mixtures, Computational biology and bioinformatics, Drug discovery

## Abstract

Fungicide mixtures are an effective strategy in delaying the development of fungicide resistance. In this research, a fixed ratio ray design method was used to generate fifty binary mixtures of five fungicides with diverse modes of action. The interaction of these mixtures was then analyzed using CA and IA models. QSAR modeling was conducted to assess their fungicidal activity through multiple linear regression (MLR), support vector machine (SVM), and artificial neural network (ANN). Most mixtures exhibited additive interaction, with the CA model proving more accurate than the IA model in predicting fungicidal activity. The MLR model showed a good linear correlation between selected theoretical descriptors by the genetic algorithm and fungicidal activity. However, both ML-based models demonstrated better predictive performance than the MLR model. The ANN model showed slightly better predictability than the SVM model, with R^2^ and R^2^_cv_ at 0.91 and 0.81, respectively. For external validation, the R^2^_test_ value was 0.845. In contrast, the SVM model had values of 0.91, 0.78, and 0.77 for the same metrics. In conclusion, the proposed ML-based model can be a valuable tool for developing potent fungicidal mixtures to delay fungicidal resistance emergence.

## Introduction

Using fungicide mixtures is a valuable strategy to delay the emergence of fungicide resistance^1–3^. Combining different modes of action can enhance control effectiveness by targeting various active sites, especially crucial when the pathogen shows resistance to specific fungicides^1–4^. It has been found that mixtures of low and high-risk fungicides can reduce the rate of fungal resistance development^3^. This can be achieved by reducing the dosage of the high-risk fungicide. Although it is always preferable to use mixtures containing fungicides with lower risk, evidence shows that mixtures comprising fungicides with high risk can also delay the development of resistance^1,3,5^. When different components are mixed together, they can interact in various ways depending on their concentration and ratios. If the combined activity of the mixture is stronger than the activity of each individual component, it is said to have a synergistic effect. On the other hand, if the mixture is less effective than the individual components, it is considered an antagonistic effect^6–9^. Therefore, it is essential to determine the optimal concentration and ratio of each component when developing effective fungicidal mixtures. This is crucial to delay resistance and reduce the use of fungicides^7,9,10^.

Toxicity prediction of pesticide mixtures is commonly done using the concentration additive (CA)^11^ and independent action (IA)^12^ models. CA is applied when the mixture components have the same mode of action (MOA), while IA is used when they have different MOAs. Despite the frequent use of these models in toxicity prediction across various chemical classes^11–13^, it is necessary to explore alternative and more precise methods for assessing the toxicity of fungicide mixtures, such as Quantitative Structure–Activity Relationship (QSAR). With QSAR, mathematical models are used to depict the relationship between the bioactivity of compounds and their physiochemical properties, known as molecular descriptors^14^. Simple QSAR methods such as multiple linear regression assume linearity in the relationship and have led to the development of many models with strong predictive capabilities. Nevertheless, in various instances, particularly when dealing with multidimensional datasets, exploring non-linear relationships is more suitable^14,15^. Recently, machine learning algorithms have been utilized in QSAR to study nonlinear structure–activity relationships^15^. Machine learning (ML) has transformed QSAR modeling with advanced tools like Artificial Neural Networks (ANNs) and Support Vector Machines (SVMs) for managing complex datasets, selecting features, and improving model accuracy^15–17^. One key benefit of using ML in QSAR modeling is its capacity to process vast amounts of data, enabling researchers to analyze complex relationships between chemical structures and biological activities^15,18,19^. Additionally, ML algorithms can automatically select the appropriate molecular descriptors that influence biological activity, reducing the risk of human error and saving time. Ultimately, this improves the overall performance and accuracy of the created models^19^. ML techniques have demonstrated remarkable accuracy in predicting the combined toxicity of nanoparticles^20^, pharmaceutical^21^, and pesticide mixtures^22^. Linear and non-linear SVM algorithms have been used to enhance the reliability of the QSAR model developed for predicting acute toxicity of binary pesticide mixtures in honey bees^22^. Back-propagation neural network (BPNN) revealed high accuracy in predicting the combined toxicity of multicomponent mixtures on *Caenorhabditis elegans*^23^.

In a recent investigation, the combined interaction of fungicides in binary mixtures was examined. Predictive models using Multiple Linear Regression (MLR) and Machine Learning-based Quantitative Structure–Activity Relationship (ML-QSAR) were developed to predict the fungicidal efficacy of these mixtures against the plant pathogenic fungus *Fusarium oxysporum* f.sp*. lycopersici*.

## Materials and methods

### Chemicals

Difenoconazole (**DF**) (purity 98%, CAS: 119446–68-3), and Tebuconazole (**TB**) (purity 99%, CAS: 107534–96-3), Thiophanate-methyl (**TM**) (purity 97%, CAS: 23564–05-8), Chlorothalonil (**CT**) (purity 98%, CAS: 1897–45-6), Iprodione (**IP**) (purity 96%, CAS: 36734–19-7) were purchased from Agricore Chemical Industry Co., Ltd. (A.C.I), China.

### Fungal strain

The fungal strain of *Fusarium oxysporum* f.sp. *lycopersici* was isolated and characterized previously in the Department of Plant Pathology of Tarbiat Modares University, Tehran, Iran.

### Signal anti-fungal activity

In order to evaluate the effectiveness of each fungicide against the mycelial growth of *F. oxysporum*, the poisoned food technique was utilized as described by Schmitz (1930)^24^. Carefully, increasing concentrations of the tested mixtures were mixed with 20–25 ml of melted warm PDA medium in a Petri dish. A 6 mm diameter agar disc of 5-day-old culture of the pathogen was aseptically transferred to the center of the Petri dish, which was then inoculated with PDA plates. Each treatment was replicated three times. A basal medium (PDA) serving as the control. The inoculated plates were incubated at a constant temperature of 25 °C, and the colony diameter was measured and recorded after 7 days. The percentage of mycelial growth inhibition was determined using the following equation:

1 $$ {\text{Mycelial}}\;{\text{growth}}\;{\text{inhibition}}\;{\text{rate}} ({\text{I}}\, \% ) = ({\text{C}} - {\text{T}})/C \times 100 $$

where C is the diameter of the fungal colony (mean) in control, and T is the diameter of the fungal colony (mean) in the presence of the synthesized compound. The respective dose–response curves and effective concentration (EC_50_) values were calculated using GraphPad Prism^25^.

### Binary mixture design

The five fungicides mentioned as (DF, TB, TM, CT, and IP) were used to form ten binary mixtures using Fixed Ratio Ray Design (FRRD) where the mixing ratio (proportions) (*p*_i_) of components was fixed across increasing mixture doses^26,27^. Five mixture rays (R1–R5) being uniformly distributed in the two-dimensional concentration space of two mixture components are designed for each binary mixture system^10^.

For a binary mixture, the p_1,* j*_ and p_2,* j*_ of components 1 and 2 in each mixture can be calculated by the following equation based on the 50% concentration effect (EC_50_, mg/L):

2 $$ {\text{p}}_{{\text{1,j}}} { = }\frac{{{\text{j}}{\text{.EC}}_{{{50}{\text{.1}}}} }}{{{\text{j}}{\text{.EC}}_{{{50}{\text{.1}}}} { + }\left( {{\text{6{-}1}}} \right){\text{.EC}}_{{{50}{\text{.2}}}} }} $$

3 $$ {\text{p}}_{{\text{2,j}}} { = }\frac{{\left( {{\text{6{-}1}}} \right){\text{.EC}}_{{{50}{\text{.2}}}} }}{{{\text{j}}{\text{.EC}}_{{{50}{\text{.1}}}} { + }\left( {{\text{6{-}1}}} \right){\text{.EC}}_{{{50}{\text{.2}}}} }} $$

where *j* refers to the series number of a ray^10^. Overall, fifty binary mixtures were deigned and test for their fungicidal activity according to the procedures mentioned on signal anti-fungal assay.

### Combined effects of mixtures (CA and *IA* models)

The expected EC_50_ of the mixtures was calculated using concentration addition model (CA)^8^ according to Eq. 4:

4 $$ {\text{ECx}}_{{{{mix}}}} { = }\left( {\mathop \sum \limits_{{\text{i = 1}}}^{{\text{n}}} \frac{{{\text{p}i}}}{{{\text{ECx}}_{{{i}}} }}} \right)^{{{\text{{-}1}}}} $$

where ECx_*mix*_ is the total concentration of the mixture provoking x% effect; ECx_*i*_ is the concentration of component *i* provoking the x% effect, when applied singly; and p*i* denotes the fraction of component *i* in the mixture.

For independent action (IA) model^7,8^, the following equation (Eq. 5) was used:

5 $$ {\text{ECx}}_{{{{mix}}}} {\text{ = 1{-}}}\mathop \prod \limits_{{\text{i = 1}}}^{{\text{n}}} \left( {{\text{1{-}piECx}}_{{{i}}} } \right) $$

To help explain the difference between observed toxicity and toxicity predicted by models, the model deviation ratio (MDR)^9^ was used as follows:

6 $$ {\text{MDR = }}\frac{{{{Expected}}}}{{{Observed }}} $$

where *Expected* is the effective concentration of the mixture that would be predicted by the model, and *Observed* is the effective concentration for the mixture obtained from toxicity testing^9^. Based on the MDR value, the types of interactions between mixture components are divided into three groups: synergistic (MDR > 2), additive (0.5 ≤ MDR ≤ 2) and antagonistic (MDR < 0.5)^9,28^.

### OSAR modeling

The relationship between the fungicidal activity of the studied mixtures (EC_50_) and their molecular descriptors was estimated via QSAR modeling to develop robust mathematical models for predicting the fungicidal activity of these mixtures. Multiple linear regression (MLR) models and machine learning ML-based QSAR models were carried out in our investigation.

#### Data set and calculation of mixture molecular descriptors

The structure of each individual compound was initially drawn and pre-optimized using molecular mechanics force fields (MM +) integrated within the HyperChem package^29^. Subsequently, the three-dimensional molecular geometries were refined using the AM1 semi-empirical quantum-chemical method with a root mean square gradient of 0.01 kcal mol^–1^. HyperChem software was employed to calculate a subset of molecular descriptors. To further optimize the geometric structure, density functional theory (DFT) was employed. Specifically, the B3LYP functional with the 6-31G** basis set was utilized to compute the quantum chemical descriptors using the Gaussian 09W software^30,31^. Additional descriptors were generated using E-Dragon 3.0 software^32^, specifically 1D-2D and 3-dimensional molecular descriptors. These descriptors were combined with the previously calculated descriptors from HyperChem and Gaussian software. The total number of molecular descriptors was reduced based on specific criteria. These criteria included: 1) Removing descriptors with a standard deviation less than 0.001, 2) Eliminating descriptors with at least one missing value, 3) Removing descriptors with constant values, and 4) Removing descriptors that were constant across all molecules or highly correlated (R ≥ 0.9)^33–35^. As a result, a total of 321 descriptors were obtained for the calculation of mixture descriptors according to Eq. (7).^36^

7 $$ {\text{D}}_{{{mix, i }}} { = }\mathop \sum \limits_{{\text{i = 1}}}^{{\text{n}}} {\text{p}}_{{{i}}} {\text{X}}_{{{i}}} $$

where D_*mix*,*i*_ represents the descriptor of a single mixture, *i* denotes the *i*th component in a mixture, and X_*i*_ stands for the descriptor of the *i*th component. The descriptors of the resulting mixtures from Eq. 7 are then refined based on the same criteria utilized for signal compounds^29–31^.

#### Genetic algorithm (GA)

The Genetic Algorithm (GA) is a search method inspired by Darwin's theory of natural selection and evolution. Due to its effectiveness and simplicity, the GA has been widely used as a promising approach for variable selection^37^. In QSAR modeling, the Genetic Algorithm can be employed to identify the most important molecular descriptors or features for predicting the bioactivity of compounds. In this particular study, the Genetic Algorithm was utilized to select the key molecular descriptors that would contribute to the development of QSAR models based on the calculated mixture descriptors mentioned earlier. The execution of the Genetic Algorithm was performed using the MATLAB software provided by MathWorks, Inc^38^.

#### K-means clustering of datasets

Following the selection of the most significant molecular descriptors using the Genetic Algorithm (GA) for QSAR modeling, the dataset was divided into a training set and a test set using the k-means classification technique. The training set consisted of 80% of the total dataset, while the remaining 20% formed the test set. To create the test set, 12 mixtures were randomly chosen from each cluster generated during the k-means process, while the remaining 38 mixtures were assigned to the training set^39^. The k-means procedure was carried out using the SPSS software.

#### Multiple linear regression (MLR) model

MLR is a technique employed for the modeling of linear relationships between dependent and independent variables^40^. In our study, the dependent variable corresponds to the compound bioactivity values, while the independent variable refers to the molecular descriptors. The regression coefficients are determined through the use of MLR, employing the least-squares curve fitting method. The regression equation can be represented as follows:

8 $$ {\text{Y }} = a_{0} + \mathop \sum \limits_{i = 1}^{n} a_{i} X_{i} $$

where Y is the dependent variable (fungicidal activity), *X*_*i*_ are the independent variables (molecular descriptors), *n* is the number of molecular descriptors, $a_{0}$ is the constant in the equation, and $a_{i}$ represent the coefficients of the descriptors. The effective concentrations (EC_50_) values of the fifty binary mixtures were converted to *p*EC_50_ level (*p*EC_50_ = –logEC_50_), and used as dependent variables, while the selected molecular descriptors by GA used as independent variables in the MLR.

### Machine learning models

In the field of QSAR modeling, machine learning algorithms provide effective tools for capturing complex non-linear relationships when linear regression falls short in representing the relationship between bioactivity and the physicochemical parameters of the molecules under study^41–43^. Different algorithms can be employed in QSAR modeling depending on the nature of the dataset used to develop the model. For instance, in this study Support Vector Machine (SVM)^44^, and Artificial Neural Network (ANN)^43^ algorithms were utilized to develop ML-QSAR models.

#### Support vector machine (SVM) regression

Support vector machine (SVM) has gained recognition as a powerful and valuable machine learning tool in QSAR studies for classification and regression purposes. Its robustness in handling non-linear relationships and strong generalization performance have made it highly respected in the field.^41,42^ In this study, the support vector machine (SVM) with was utilized to develop a machine learning-quantitative structure–activity relationship (ML-QSAR) model using the same four molecular descriptors as the multiple linear regression (MLR) model. Bayesian optimization was performed on the hyperparameters: kernel function, box constraint level, and kernel scale. The accuracy of the predictions was evaluated using the coefficient of determination (R^2^) and the mean square of prediction (MSE). The calculations were performed using the MATLAB software provided by MathWorks, Inc^38^.

#### Multi-layer perceptron artificial neural network method (MLP-ANN)

The Multilayer perceptron artificial neural network (MLP-ANN) is a machine learning technique that offers a promising approach for addressing nonlinear problems^47,48^. The MLP network consists of one or more hidden layers, and the number of neurons in these hidden layers depends on the input and output datasets. In this particular study, the MLP-ANN was utilized to validate the accuracy of the selected molecular descriptors obtained from the MLR model. These descriptors were utilized as inputs for training the MLP-ANN network. The back-propagation (BP) algorithm was employed in the MLP-ANN for learning and updating the network's weights. Similar to Support Vector Machines (SVM), the prediction accuracy of the MLP-ANN was assessed. The analysis of the artificial neural network was conducted using the MATLAB software^49^.

### Internal validation of the generated models

#### Leave many out cross validation (LMO-CV)

Our study aimed to assess the reliability of statistical models by employing cross-validation, a commonly used statistical technique for internal validation. Cross-validation involves repeatedly withholding different proportions of the training set as a validation set to evaluate the predictive power of the generated QSAR models. Among the cross-validation techniques commonly employed in QSAR studies, leave-one-out cross-validation (LOO-CV) and leave many out cross-validation (LMO-CV) are the most popular. In LOO-CV, one compound is designated as the validation set in each step, while the remaining compounds serve as the training set. This process is repeated for each compound, resulting in a total of n iterations, where n represents the number of compounds. The LMO-CV method is employed by selecting M compounds as the validation set while using the remaining compounds as the training set. To evaluate the predictive performance of the GA-MLR models, the internal validation technique of LMO-CV was utilized, where 20% of the training dataset was designated as the validation set. The training process was repeated 10^+4^ times. A model is considered internally robust in its predictions if it achieves a Cross-validated correlation coefficient (R^2^_cv_) greater than 0.5^50–52^.

#### Y-randomization test (Y-scrambling test)

Y-randomization (Y-scrambling test) is a method used to assess the influence of chance in fitting data with generated models. Its purpose is to investigate and eliminate any random associations between descriptors and their corresponding biological activities within the model initially obtained through the MLR technique^53^. In this process, the dependent variable vector is randomly shuffled, while the independent variables remain unchanged. A new QSAR model is then developed using the shuffled data. The Y-randomization test considers the QSAR model valid and not a result of chance when the average random coefficient of determination (R^2^) and cross-validated R^2^ (R^2^_cv_) values of the randomly generated models are lower than the coefficient of determination (R^2^) of the original model^54^.

#### Applicability domain

According to The OECD principles, it is imperative that QSAR models incorporate a specified domain of applicability^55^. The assessment of the applicability domain assists in understanding if the constructed QSAR model can be applied to any given group of compounds. The applicability domain of the QSAR model is defined as a theoretical region within the chemical space of descriptors and the predicted activity^56^. This region encompasses the molecules for which their bioactivity is reliably estimated through the use of a quantitative structure–activity relationship (QSAR) model. However, the molecules for which their bioactivities are not accurately predicted are positioned outside of this domain, commonly referred to as outliers^56,57^.

The leverage approach is one of the most frequently used methods for defining the AD^56,58^. This can be achieved by utilizing the following formula:

9 $$ {{h}}_{{{i}}} {{ = x}}_{{{i}}}^{{{T}}} \left( {{{x}}^{{{T}}} {{x}}} \right)^{{{{{-}1}}}} {{x}}_{{{i}}} { }\left( {{{i = 1, 2, 3 \ldots \ldots n}}} \right) $$

where *h*_*i*_ represents the leverage of a compound, *x*_*i*_ represents the descriptor row vector of the studied compound, and *x* represents the whole matrix of the descriptor values of compounds in the training set. *T* is matrix or vector-transposed symbol.

### External validation

The performance of the developed models was additionally evaluated on an external test set, which was created using the k-means clustering method mentioned earlier. This test set comprises 12 mixtures, accounting for 20% of the total mixtures, that were not used in the development and training of the models. The model's ability to predict the fungicidal activity of the external test set was assessed using two metrics: R^2^_test_ and MSE_test_. If the R^2^_test_ value is greater than 0.5, the model is considered to be acceptable in terms of its predictive performance^52^.

## Results and discussion

### Fungicidal activity of tested fungicides and binary mixtures

**Figure 1:**
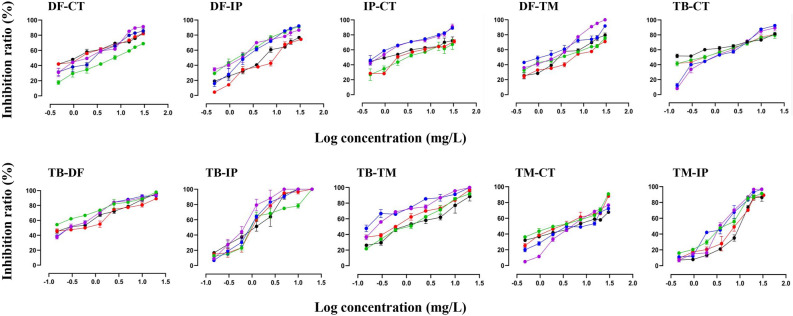
Dose response carvers of the studied mixtures. Black curve refers to the Ray1, Red to Ray 2, Green to Ray 3, Blue to Ray 4, and Purple to Ray 5 in each binary mixture.

The five fungicides mentioned were assessed for their fungicidal efficacy against the plant pathogenic fungi *F. oxysporum*. The EC_50_ values for each fungicide are presented in Table 1. It was noted that the azole fungicide TB demonstrated superior fungicidal effectiveness, followed by IP and TM. In contrast, the azole fungicide DF showed notably lower activity compared to the other compounds tested. The binary combinations of the tested fungicides showed a variety of interactions between the compounds. The effective concentrations (EC_50_) and component ratios (p_1*, j*_, and p_2*, j*_) for the fifty binary mixtures designed and tested using the Fixed Ratio Ray Design (FRRD) were detailed in Table 2. The dose–response curves of the fifty tested mixtures are illustrated in Fig. 1.

**Table 1:** The observed effective concentrations (EC_50_) of the tested fungicides.

Fungicide	EC_50_ (mg/L)	EC_50, lower_	EC_50, upper_
TM	2.953	2.405	3.631
CT	4.182	2.598	6.615
DF	6.429	5.047	8.240
TB	0.58	0.485	0.698
IP	2.296	1.204	4.907

**Table 2:** The observed effective concentrations (EC_50_) of the tested mixtures using FRRD.

No	Mixture ray	p_1,* j*_	p_2,* j*_	EC_50, obs._ (mg/L)	*p*EC_50_	EC_50, lower_	EC_50, upper_
1	TM-CT (R1)	0.12	0.88	6.554	− 0.8165	3.676	12.46
2	TM-CT (R2)	0.26	0.74	4.658	− 0.6682	1.341	6.59
3	TM-CT (R3)	0.41	0.59	4.379	− 0.6414	1.887	7.26
4	TM-CT (R4)	0.59	0.41	2.798	− 0.4468	1.428	5.961
5	TM-CT (R5)	0.78	0.22	2.307	− 0.3630	0.9416	3.113
6	TM-IP (R1)	0.2	0.8	9.65	− 0.9845	8.643	11.02
7	TM-IP (R2)	0.39	0.61	7.5	− 0.8751	5.689	9.035
8	TM-IP (R3)	0.56	0.44	7	− 0.8451	5.148	8.53
9	TM-IP (R4)	0.72	0.28	4.681	− 0.6703	3.002	7.413
10	TM-IP (R5)	0.87	0.13	4.482	− 0.6515	3.835	5.374
11	IP-CT (R1)	0.1	0.9	2.97	− 0.4728	1.23	7.44
12	IP-CT (R2)	0.22	0.78	1.748	− 0.2425	0.2335	2.383
13	IP-CT (R3)	0.35	0.65	1.704	− 0.2315	0.6882	3.877
14	IP-CT (R4)	0.52	0.48	1.449	− 0.1611	0.5608	3.416
15	IP-CT (R5)	0.73	0.27	1.319	− 0.1202	0.4972	3.191
16	DF-TM (R1)	0.92	0.08	3.428	− 0.5350	2.155	5.548
17	DF-TM (R2)	0.81	0.19	5.427	− 0.7346	2.898	8.523
18	DF-TM (R3)	0.69	0.31	6.812	− 0.8333	2.979	11.76
19	DF-TM (R4)	0.52	0.48	7.197	− 0.8572	3.009	12.351
20	DF-TM (R5)	0.3	0.7	7.442	− 0.8717	5.98	9.328
21	DF-CT (R1)	0.24	0.76	2.996	− 0.4765	1.618	5.65
22	DF-CT (R2)	0.43	0.57	4.137	− 0.6167	2.607	6.719
23	DF-CT (R3)	0.61	0.39	4.672	− 0.6695	2.721	8.168
24	DF-CT (R4)	0.75	0.25	5.143	− 0.7112	3.432	7.836
25	DF-CT (R5)	0.88	0.12	7.783	− 0.8911	4.212	14.9
26	DF-IP (R1)	0.36	0.64	5.554	− 0.7446	3.809	8.237
27	DF-IP (R2)	0.58	0.42	4.902	− 0.6904	2.535	9.428
28	DF-IP (R3)	0.74	0.26	2.308	− 0.3632	1.707	3.104
29	DF-IP (R4)	0.85	0.15	2.178	− 0.3381	1.512	3.117
30	DF-IP (R5)	0.93	0.07	2.076	− 0.3172	1.277	3.336
31	TB-CT (R1)	0.03	0.97	3.323	− 0.5215	1.354	8.29
32	TB-CT (R2)	0.07	0.93	2.593	− 0.4138	1.846	5.94
33	TB-CT (R3)	0.11	0.88	2.123	− 0.3269	1.356	3.33
34	TB-CT (R4)	0.15	0.78	1.752	− 0.2435	0.6678	4.123
35	TB-CT (R5)	0.19	0.59	0.7	0.1549	0.3088	1.484
36	TB-DF (R1)	0.02	0.98	2.283	− 0.3585	1.483	3.548
37	TB-DF (R2)	0.04	0.96	1.896	− 0.2778	1.52	2.643
38	TB-DF (R3)	0.08	0.92	1.35	− 0.1303	0.7783	2.353
39	TB-DF (R4)	0.15	0.85	0.6773	0.1692	0.4953	0.913
40	TB-DF (R5)	0.31	0.69	0.6345	0.1976	0.4355	0.9075
41	TE-TM (R1)	0.05	0.95	2.208	− 0.3440	0.8377	6.026
42	TE-TM (R2)	0.11	0.89	1.909	− 0.2808	1.142	3.259
43	TE-TM (R3)	0.2	0.8	1.867	− 0.2711	1.184	2.919
44	TE-TM (R4)	0.34	0.66	0.8243	0.0839	0.3323	1.833
45	TE-TM (R5)	0.56	0.44	0.5012	0.3000	0.1825	1.238
46	TE-IP (R1)	0.05	0.95	1.782	− 0.2509	1.157	2.721
47	TE-IP (R2)	0.11	0.89	1.132	− 0.0538	0.7632	1.668
48	TE-IP (R3)	0.2	0.8	0.9769	0.0101	0.5453	1.753
49	TE-IP (R4)	0.34	0.66	0.7815	0.1071	0.6014	1.008
50	TE-IP (R5)	0.56	0.44	0.423	0.3737	0.2729	0.6317

The MDR values for the CA model ranged from 0.25 (Mix 6) to 3.78 (Mix 39), whereas for the IA model, the values varied between 0.001 (Mix 20) and 7.49 (Mix 39) as shown in Table S1. In the case of the CA model, 74% of the mixtures (37 in total) displayed additive interaction with MDR values from 0.5 to 2. Conversely, 8% (4 mixtures) and 18% (9 mixtures) exhibited antagonistic and synergistic interactions, respectively. In the IA model, 40% (20 mixtures) of the combinations exhibited additive interaction, with 36% (18 mixtures) showing antagonistic interactions, and 24% (12 mixtures) displaying synergistic interactions. In various mixtures (e.g. 35, 38, 39, and 50), MDR values for the IA model were notably higher than the CA model, suggesting that the IA model might tend to underestimate the fungicidal activity of the mixture compared to the CA model. Our findings support prior research results^9,59,60^. Compared to the IA model, the CA model exhibited a higher proportion of MDR values close to 1. Additionally, the CA model had a coefficient of determination (R^2^) and mean square error (MSE) of 0.17 and 4.45, respectively. In contrast, the IA model showed no correlation between the observed and estimated EC_50_ values, indicating that the CA model might offer more precise estimates of the fungicidal activity of the analyzed mixtures. Zhang and Liu (2015)^60^ showed that the CA model provides a good evaluation of the combined toxicity of organophosphorus pesticides, but the IA model tends to underestimate the joint toxicity of the compounds under study. Comparable results were shown when using CA and IA models to estimate the combined toxicity of medicines and chlorophenols to freshwater algae^59^.

### QSAR modeling

Through QSAR modeling, the relationship between the fungicidal activity and physicochemical parameters of the investigated mixtures has been evaluated. The genetic algorithm (GA) was utilized to determine the key molecular descriptors that impact the fungicidal activity of the mixtures, calculated according to specified criteria in the materials and methods section. Four molecular descriptors ATS3m (Broto-Moreau autocorrelation of lag 3 weighted by mass from 2D autocorrelations descriptors), MLOGP (Moriguchi octanol–water partition coefficient from molecular descriptors)^32^, E_*LUMO*_ (Least unoccupied molecular orbital from quantum chemical descriptors), and electronegativity (*x*) from quantum chemical descriptors were chosen to develop the QSAR models. For each of the fifty mixtures, the values of these descriptors were calculated (Table S2). The K-means classification was then used to split the entire dataset into train and test sets. Thus, to create the models, the following mixed rays: 3, 10, 14, 20, 23, 25, 27, 32, 36, 41, 46, and 50, were used as test set, while the remaining rays were utilized as train set. The *p*EC_50_ values of the mixtures and the four selected molecular descriptors by GA were used as input for QSAR model.

### MLR model

To evaluate the linearity of the relationship between the mixtures *p*EC_50_ and the selected molecular descriptors using GA, the multiple linear regression method was used, leading to the following model (Eq. 10):

10 $$ p{\text{EC}}_{50} = \, \left( {{-}1.199 \, \pm \, 0.878} \right) \, +_{{}} \left( {{-}0.1434 \, \pm \, 0.0214} \right){\text{ATS}}3m + \, \left( {2.273 \, \pm \, 0.393} \right){\text{MLOGP}} + \, \left( {1.216 \, \pm \, 0.307} \right){\text{E}}_{LUMO} + \, \left( {1.180 \, \pm \, 0.382} \right)x $$

where n = 38, R^2^_(train)_ = 0.625, R^2^_(adj.)_ = 0.58, MSE _(train)_ = 0.044, P < 0.001, R^2^_(test) =_ 0.551, MSE _(test)_ = 0.052, R^2^_*cv*_ = 0.55.

Equation 10 revealed a good linear correlation between the four selected descriptors by GA and the fungicidal activity of the mixtures (*p*EC_50_). The statistical parameters R^2^_(train)_ = 0.63, MSE _(train)_ = 0.044, R^2^_*cv*_ = 0.55, and a *p*-value below 0.05 demonstrates that the MLR model is statistically acceptable.

**Figure 2:**
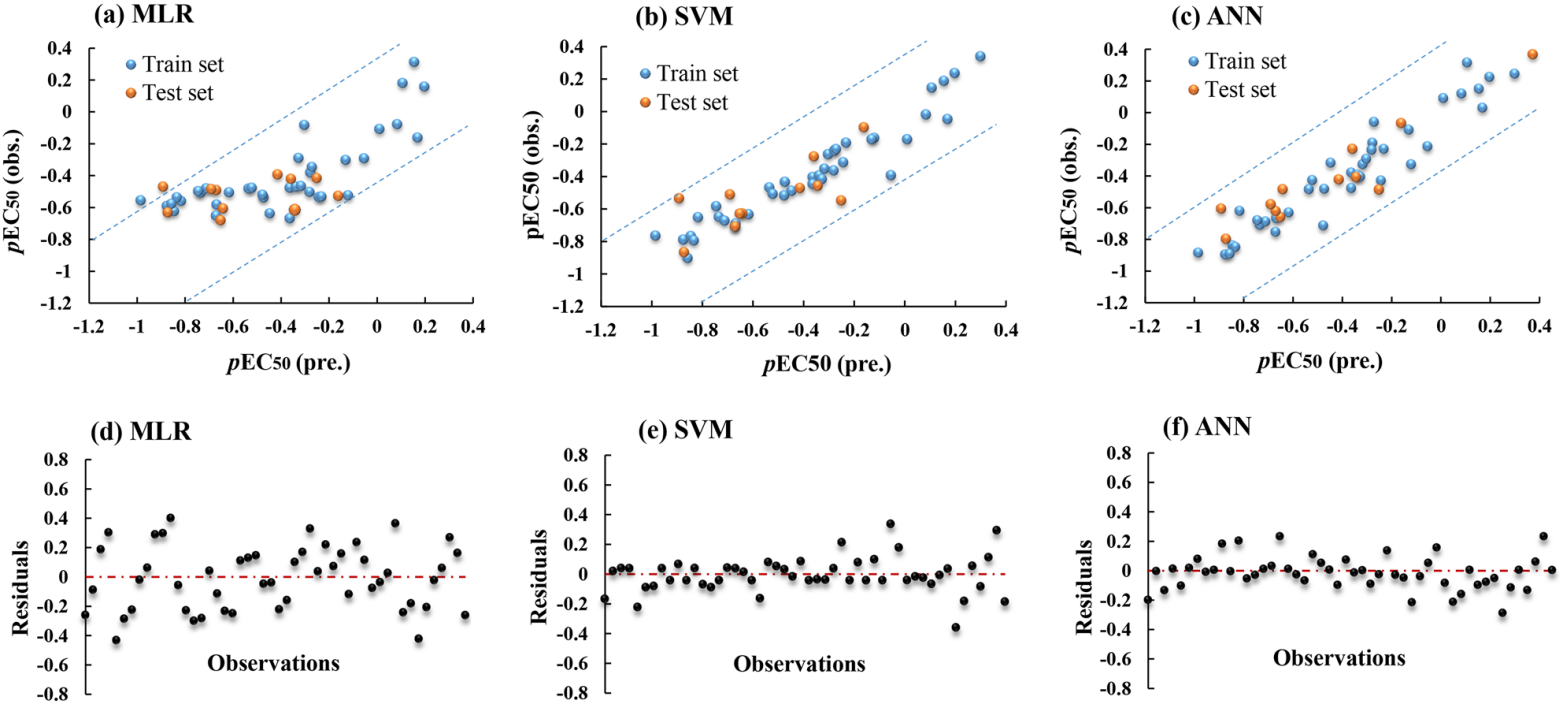
Experimental *vs* predicted *p*EC_50_ values of (**a**) MLR, (**b**) SVM, and (**c**) ANN. The plot of residulas destribution of (**d**) MLR, (**e**) SVM, and (**f**) ANN.

The cross-validation correlation coefficient R^2^_*cv*_ = 0.55 > 0.5, indicates that the MLR model is accurate in predicting the fungicidal activity of data excluded from the train set. The distribution of the experimental and predicated *p*EC_50_ values through MLR model is shown in Fig. 2. A good correlation between experimental and predicated *p*EC_50_ values can be detected.

### Y-randomization (Y-scrambling) test for GA-MLR model

**Figure 3:**
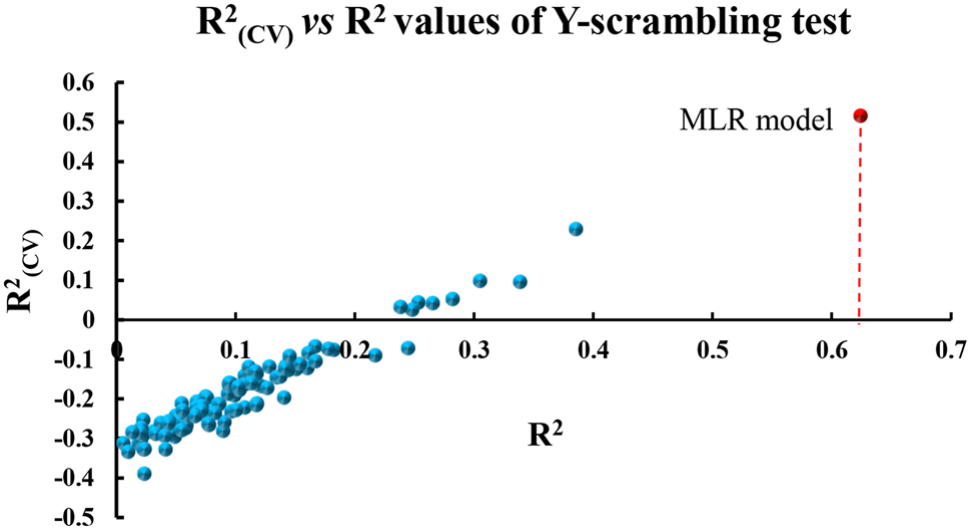
Y-Randomization (Y-scrambling) test results for the MLR model.

The dependent variable (*p*EC_50_) vector is randomly scrambled with the goal of ensuring the robustness of the MLR model. Four chemical descriptors, the independent variable, remain unchanged while 100 new models are created. The R^2^ and R^2^_*CV*_ values of the generated models were lower than the original values obtained from the original MLR model (R, R^2^, R^2^_*CV*_) as shown in Fig. 3. The Y-randomization test indicates that the model obtained in Eq. (10) is robust and the *p*EC_50_ values predicted by the MLR model (Table 3) are not due to chance.

**Table 3:** EC_50_ and residual values predicted by generated QSAR models.

No	Mixture ray	*p*EC_50, obs_	MLR		SVM		ANN	
			*p*EC_50_	Residual	*p*EC_50_	Residual	*p*EC_50_	Residual
Train set								
1	TM-CT (R1)	− 0.8165	− 0.5572	− 0.2593	− 0.6507	− 0.1658	− 0.6194	− 0.1971
2	TM-CT (R2)	− 0.6682	− 0.5804	− 0.0878	− 0.6897	0.0215	− 0.6660	− 0.0022
4	TM-CT (R4)	− 0.4468	− 0.6351	0.1882	− 0.4878	0.0409	− 0.3150	− 0.1319
5	TM-CT (R5)	− 0.3630	− 0.6665	0.3035	− 0.4030	0.0399	− 0.3772	0.0141
6	TM-IP (R1)	− 0.9845	− 0.5541	− 0.4304	− 0.7631	− 0.2214	− 0.8837	− 0.1008
7	TM-IP (R2)	− 0.8751	− 0.5895	− 0.2856	− 0.7875	− 0.0875	− 0.8951	0.0201
8	TM-IP (R3)	− 0.8451	− 0.6211	− 0.2240	− 0.7660	− 0.0791	− 0.7518	0.0815
9	TM-IP (R4)	− 0.6703	− 0.6509	− 0.0194	− 0.7101	0.0398	− 0.8377	− 0.0074
11	IP-CT (R1)	− 0.4728	− 0.5352	0.0625	− 0.4326	− 0.0402	− 0.4800	0.0072
12	IP-CT (R2)	− 0.2425	− 0.5328	0.2903	− 0.3112	0.0687	− 0.4272	0.1847
13	IP-CT (R3)	− 0.2315	− 0.5302	0.2987	− 0.1913	− 0.0401	− 0.2280	− 0.0035
15	IP-CT (R5)	− 0.1202	− 0.5224	0.4022	− 0.1611	0.0409	− 0.3251	0.2048
16	DF-TM (R1)	− 0.5350	− 0.4793	− 0.0558	− 0.4671	− 0.0680	− 0.4832	− 0.0518
17	DF-TM (R2)	− 0.7346	− 0.5060	− 0.2285	− 0.6475	− 0.0871	− 0.7074	− 0.0272
18	DF-TM (R3)	− 0.8333	− 0.5352	− 0.2981	− 0.7931	− 0.0402	− 0.8461	0.0129
19	DF-TM (R4)	− 0.8572	− 0.5766	− 0.2806	− 0.9009	0.0437	− 0.8913	0.0341
21	DF-CT (R1)	− 0.4765	− 0.5187	0.0421	− 0.5166	0.0401	− 0.7114	0.2348
22	DF-CT (R2)	− 0.6167	− 0.5040	− 0.1127	− 0.6321	0.0154	− 0.6297	0.0130
24	DF-CT (R4)	− 0.7112	− 0.4792	− 0.2320	− 0.6709	− 0.0403	− 0.6869	− 0.0244
26	DF-IP (R1)	− 0.7446	− 0.4964	− 0.2482	− 0.5827	− 0.1620	− 0.6788	− 0.0658
28	DF-IP (R3)	− 0.3632	− 0.4747	0.1114	− 0.4454	0.0821	− 0.4752	0.1119
29	DF-IP (R4)	− 0.3381	− 0.4684	0.1303	− 0.3930	0.0549	− 0.3918	0.0538
30	DF-IP (R5)	− 0.3172	− 0.4638	0.1466	− 0.3506	0.0334	− 0.3254	0.0081
31	TB-CT (R1)	− 0.5215	− 0.4750	− 0.0465	− 0.5062	− 0.0153	− 0.4255	− 0.0960
33	TB-CT (R3)	− 0.3269	− 0.2882	− 0.0388	− 0.4146	0.0876	− 0.4028	0.0759
34	TB-CT (R4)	− 0.3023	− 0.0806	− 0.2217	− 0.2623	− 0.0400	− 0.2910	− 0.0113
35	TB-CT (R5)	0.1549	0.3138	− 0.1589	0.1894	− 0.0345	0.1515	0.0034
37	TB-DF (R2)	− 0.2778	− 0.3799	0.1021	− 0.2409	− 0.0369	− 0.1893	− 0.0885
38	TB-DF (R3)	− 0.1303	− 0.3000	0.1696	− 0.1703	0.0400	− 0.1070	− 0.0234
39	TB-DF (R4)	0.1692	− 0.1601	0.3293	− 0.0454	0.2146	0.0312	0.1380
40	TB-DF (R5)	0.1976	0.1596	0.0379	0.2377	− 0.0401	0.2256	− 0.0281
42	TB-TM (R2)	− 0.2808	− 0.5013	0.2205	− 0.3616	0.0808	− 0.2349	− 0.0460
43	TB-TM (R3)	− 0.2711	− 0.3444	0.0732	− 0.2310	− 0.0402	− 0.0582	− 0.2130
44	TB-TM (R4)	0.0839	− 0.0754	0.1593	− 0.0171	0.1010	0.1213	− 0.0373
45	TB-TM (R5)	0.3000	0.4177	− 0.1177	0.3407	− 0.0407	0.2460	0.0540
47	TB-IP (R2)	− 0.0538	− 0.2908	0.2370	− 0.3919	0.3381	− 0.2124	0.1586
48	TB-IP (R3)	0.0101	− 0.1059	0.1160	− 0.1696	0.1798	0.0925	− 0.0823
49	TB-IP (R4)	0.1071	0.1819	− 0.0748	0.1470	− 0.0399	0.3173	− 0.2103
Test set								
3	TM-CT (R3)	− 0.6414	− 0.6052	− 0.0361	− 0.6267	− 0.0147	− 0.4831	− 0.1582
10	TM-IP (R5)	− 0.6515	− 0.6788	0.0273	− 0.6282	− 0.0233	− 0.6584	0.0069
14	IP-CT (R4)	− 0.1611	− 0.5267	0.3656	− 0.0958	− 0.0653	− 0.0652	− 0.0959
20	DF-TM (R5)	− 0.8717	− 0.6301	− 0.2416	− 0.8661	− 0.0056	− 0.7962	− 0.0755
23	DF-CT (R3)	− 0.6695	− 0.4900	− 0.1795	− 0.7065	0.0370	− 0.6203	− 0.0492
25	DF-CT (R5)	− 0.8911	− 0.4691	− 0.4220	− 0.5335	− 0.3576	− 0.6050	− 0.2861
27	DF-IP (R2)	− 0.6904	− 0.4838	− 0.2066	− 0.5104	− 0.1800	− 0.5779	− 0.1125
32	TB-CT (R2)	− 0.4138	− 0.3920	− 0.0218	− 0.4700	0.0562	− 0.4209	0.0071
36	TB-DF (R1)	− 0.3585	− 0.4199	0.0614	− 0.2758	− 0.0827	− 0.2280	− 0.1305
41	TB-TM (R1)	− 0.3440	− 0.6134	0.2694	− 0.4574	0.1134	− 0.4061	0.0621
46	TB-IP (R1)	− 0.2509	− 0.4142	0.1632	− 0.5470	0.2961	− 0.4852	0.2343
50	TB-IP (R5)	0.3737	0.6341	− 0.2604	0.5577	− 0.1841	0.3686	0.0051

#### Applicability domain analysis of MLR model

**Figure 4:**
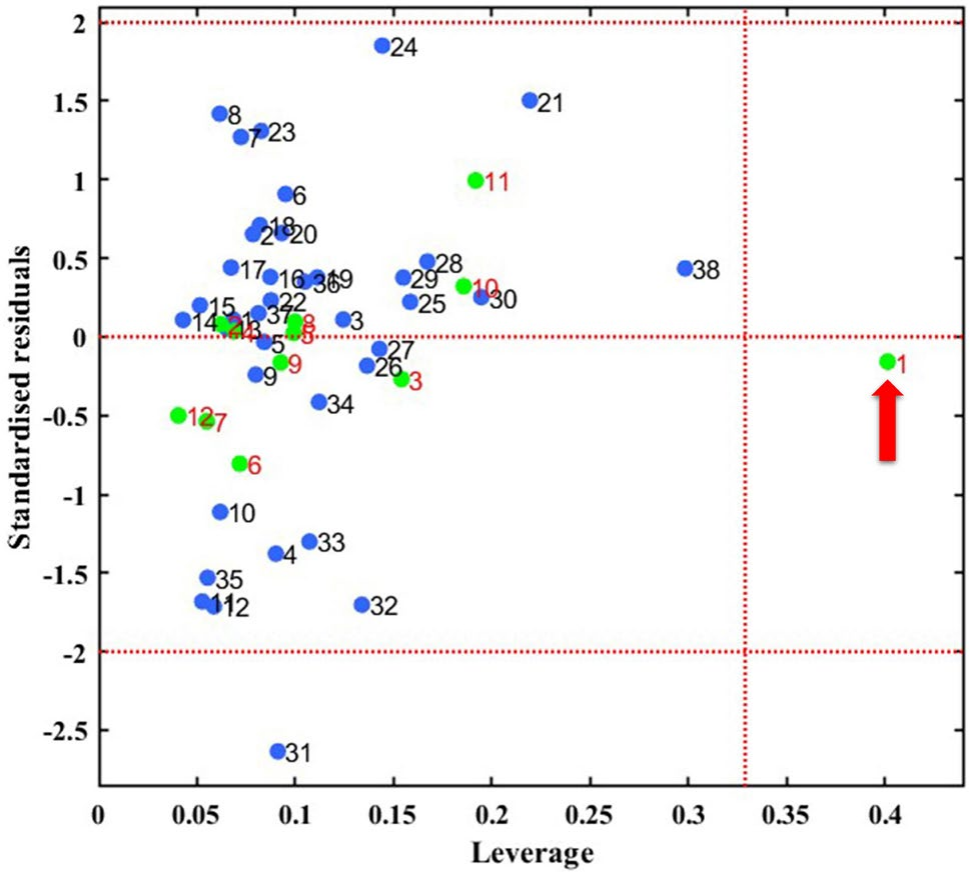
Williams plots of the QSAR models developed by the MLR. Blue circles represent the train set while the green circles represent the test set. Red arrow refers to the mixture completely outside the AD.

Figure 4 illustrates the Williams plot of AD analysis. It illustrates the connection between the standardized cross-validated residuals and the leverage values (*h*_*i*_) for the examined mixtures. Any mixture that exhibits standardized residuals in prediction greater than three standard deviation units (*r*_i_ > 3σ) or a leverage value greater than the threshold value (*h*_*i*_ > h^*^) is considered an outlier of the AD^57^. As depicted in Fig. 4, mixture 1 from the test set can be identified as an outlier due to its high leverage value (*h*_*i*_ > 0.328). Consequently, this particular mixture is considered distant from the other mixtures in the test set and lies outside the AD space, as its leverage value surpasses the vertical leverage threshold line. Similarly, mixture 31 from the training set is also incorrectly predicted due to its standardized residuals in prediction being greater or lower than ± 2. However, it still falls within the cutoff leverage value and hence belongs to the model AD. Therefore, the prediction for mixture 31 should be accepted. However, the presence of mixture 1 from the test set outside the AP (Acceptance Probability) justifies the low external predictive power of the MLR model.

### SVM-QSAR regression model

To enhance the correlation between the fungicidal activity and the selected molecular descriptors obtained by the MLR model, the four chosen descriptors were employed as inputs for the SVM regression modeling. Support vector regression has been utilized in various regression problems^61^. Gaussian kernel function, box constraint level of 904.61, and kernel scale of 6.479 were chosen as the optimal hyperparameters through Bayesian optimization. The mean square error (MSE) is 0.01, with the coefficient of determination (R^2^) and R^2^_cv_ being 0.91 and 0.78, respectively (Table 4), indicating the superior predictive ability of the SVM-based QSAR model over the MLR model. It can be inferred that the predicted values align well with the experimental values, as shown in Fig. 2.

**Table 4:** Comparison of MLR, SVM and ANN generated models.

Model	Train set				Test set	
	R^2^	MSE	R^2^_cv_	MSE_cv_	R^2^_test_	MSE_test_
MLR	0.625	0.044	0.55	0.056	0.551	0.052
SVM	0.91	0.01	0.78	0.025	0.77	0.026
ANN	0.91	0.01	0.81	0.017	0.845	0.018

Support vector regression is presently widely employed for QSAR modeling in various fields like solubility of solid solutes^62^, dipeptide datasets^44^, Materials^63^, and drug design and screening^64^. The high predictive capability of the SVM regression model in our study renders it potentially useful as a powerful tool for designing novel fungicide mixtures to address the resistance problem encountered in modern agriculture.

### ANN-QSAR model

Multi-layer perceptron networks are the most widely used feedforward artificial neural networks for solving nonlinear relationships in QSAR studies^65^. The same input used for the SVM-QSAR model was also used for the ANN-QSAR model. In this study, a backpropagation algorithm was utilized for training the ANN-QSAR model. The number of neurons in the hidden layer was varied from 1 to 30 in order to determine the optimal number of neurons based on the root mean square error values. It was determined that the network possessed an architecture of 4/4/1 (with one hidden layer consisting of 4 neurons) exhibited the most optimal performance for our inputs. The dataset was divided into three subsets in a random manner for the ANN model: 65% for the training set, 15% for the testing set, and 20% for the validation set. The training set showed an R^2^ value of 0.91 and an MSE value of 0.011, indicating the excellent predictability of the non-linear model generated by ANN. The R^2^_cv_ value was 0.81, which surpassed the other models (Table 4). The MLP-ANN has been successfully employed as a powerful modeling tool for various modeling and predicting studies, such as predicting the normal boiling point temperature and relative liquid density^66^, predicting protein structure^65^, and the bioactivity of drugs and pesticides^67–69^. The statistical parameters of the developed ANN-QSAR model in this study demonstrated the high performance of the MLP-ANN in predicting the fungicidal activity of the mixtures.

There is no agreement on a universally accepted optimal algorithm for model development in field of QSAR and QSPR^70^. A comparative analysis of the statistical parameters obtained from the generated QSAR models in this investigation revealed that both ANN and SVM models exhibited a high level of predictive performance in comparison to the MLR model, as evidenced by the MSE and R^2^_cv_ values (Table 4). The residuals of both the SVM and ANN models were found to be distributed randomly on either side of zero and closer to the zero line when compared to the MLR model. This observation suggests the superiority of the ML-based QSAR model over the classic MLR model. However, the high R^2^_cv_ value indicated that the ANN-QSAR model achieved higher accuracy than the SVM model, highlighting the robustness of the ANN model even when excluding a portion of the training set data as the validation set during model development. Furthermore, the results of the external validation demonstrated the superiority of the ANN model over both the MLR and SVM models, based on the R^2^_test_ and MSE_test_ values as indicated in Table 4.

## Conclusions

In the current investigation, the fixed ratio Ray design was employed to formulate 50 potential binary mixtures using 5 fungicides extensively utilized in the agricultural field. The interaction type of the mixture components was determined through the utilization of the CA and IA models. Various interactions were observed among the fungicides, with the majority being additive based on the models utilized. Additionally, QSAR models were developed to predict the fungicidal activity of these mixtures. The ML-based QSAR models SVM and ANN displayed a high level of predictive performance when compared to the MLR model. Nevertheless, the ANN-QSAR model achieved greater accuracy than the SVM model. This study emphasizes the potential use of these models in developing improved fungicide mixtures for agriculture purposes. The findings could have a significant influence on managing plant disease resistance.

## Supplementary Information

Supplementary Information.

## Data Availability

The authors confirm that the data supporting the findings of this study are available within the article and its supplementary materials.

## References

[1] Brent, K.J., & Hollomon, D.W., 1995. Fungicide resistance in crop pathogens: How can it be managed?. 48.

[2] Bolton, N. J. E., & Smith, J. M. 1988. Strategies to combat fungicide resistance in barley powdery mildew. in *British Crop Protection Conference, Pests & Diseases* 367–372.

[3] Van Den Bosch, F., Oliver, R., Van Den Berg, F. & Paveley, N. Governing principles can guide fungicide-resistance management tactics. *Annu Rev Phytopathol.* **52**, 175–195 (2014). 24848413 10.1146/annurev-phyto-102313-050158

[4] Oliver, R.P., & Hewitt, H.G. (2014). Fungicides in crop protection. Cabi. 200pp.

[5] Birch, C. P. D., & Shaw, M. W. When can reduced doses and pesticide mixtures delay the build-up of pesticide resistance? A mathematical model. *J. Appl. Ecol. *(1997): 1032–1042

[6] Campitelli, M., Zeineddine, N., Samaha, G. & Maslak, S. Combination antifungal therapy: A review of current data. *J. Clin. Med. Res.* **9**(6), 451 (2017). 28496543 10.14740/jocmr2992w PMC5412516

[7] Altenburger, R., Nendza, M. & Schüürmann, G. Mixture toxicity and its modeling by quantitative structure-activity relationships. *Environ. Toxicol. Chem. Int. J.* **22**(8), 1900–1915 (2003). 10.1897/01-386 12924589

[8] Altenburger, R., Walter, H. & Grote, M. What contributes to the combined effect of a complex mixture?. *Environ. Sci. Technol.* **38**(23), 6353–6362 (2004). 15597892 10.1021/es049528k

[9] Belden, J. B., Gilliom, R. J. & Lydy, M. J. How well can we predict the toxicity of pesticide mixtures to aquatic life?. *Integrated Environ. Assess. Manag. Int. J.* **3**(3), 364–372 (2007). 17695109

[10] Liu, L., Liu, S. S., Yu, M. & Chen, F. Application of the combination index integrated with confidence intervals to study the toxicological interactions of antibiotics and pesticides in Vibrio qinghaiensis sp-Q67. *Environ. Toxicol. Pharmacol.* **39**(1), 447–456 (2015). 25589171 10.1016/j.etap.2014.12.013

[11] Loewe, S. T. Effect of combinations: mathematical basis of problem. *Arch. Exp. Pathol. Pharmakol.* **114**, 313–326 (1926).

[12] Bliss, C. I. The toxicity of poisons applied jointly 1. *Ann. Appl. Biol.* **26**(3), 585–615 (1939).

[13] Lydy, M., Belden, J., Wheelock, C., Hammock, B., Denton, D., 2004. Challenges in regulating pesticide mixtures. *Ecol. Soc.**9*(6).

[14] Todeschini, R. & Consonni, V. *Handbook of Molecular Descriptors* 688 (Wiley, New York, 2008).

[15] Keyvanpour, M. R. & Shirzad, M. B. An analysis of QSAR research based on machine learning concepts. *Curr. Drug Discov. Technol.* **18**(1), 17–30 (2021). 32178612 10.2174/1570163817666200316104404

[16] Czermiński, R., Yasri, A. & Hartsough, D. Use of support vector machine in pattern classification: Application to QSAR studies. *Quant. Struct. Activity Relation.* **20**(3), 227–240 (2001).

[17] Rumelhart, D. E., Hinton, G. E. & Williams, R. J. Learning representations by back-propagating errors. *Nature* **323**(6088), 533–536 (1986).

[18] Johnson, S. R. The trouble with QSAR (or how I learned to stop worrying and embrace fallacy). *J. Chem. Inf. Model.* **48**(1), 25–26 (2008). 18161959 10.1021/ci700332k

[19] Zhang, L., Tan, J., Han, D. & Zhu, H. From machine learning to deep learning: progress in machine intelligence for rational drug discovery. *Drug Discovery Today* **22**(11), 1680–1685 (2017). 28881183 10.1016/j.drudis.2017.08.010

[20] Zhang, F., Wang, Z., Peijnenburg, W. J. & Vijver, M. G. Machine learning-driven QSAR models for predicting the mixture toxicity of nanoparticles. *Environ. Int.* **177**, 108025 (2023). 37329761 10.1016/j.envint.2023.108025

[21] Chatterjee, M. & Roy, K. Chemical similarity and machine learning-based approaches for the prediction of aquatic toxicity of binary and multicomponent pharmaceutical and pesticide mixtures against *Aliivibrio fischeri*. *Chemosphere* **308**, 136463 (2022). 36122748 10.1016/j.chemosphere.2022.136463

[22] Chatterjee, M. *et al.* Machine learning-based q-RASAR modeling to predict acute contact toxicity of binary organic pesticide mixtures in honey bees. *J. Hazardous Mater.* **460**, 132358 (2023). 10.1016/j.jhazmat.2023.132358 37634379

[23] Wang, Z. J., Liu, S. S., Feng, L. & Xu, Y. Q. BNNmix: A new approach for predicting the mixture toxicity of multiple components based on the back-propagation neural network. *Sci. Total Environ.* **738**, 140317 (2020). 32806371 10.1016/j.scitotenv.2020.140317

[24] Schmitz, H. Poisoned food technique. *Ind. Eng. Chem. Anal. Edition* **2**(4), 361–363 (1930).

[25] GraphPad Prism (Version 7) [Computer software]. La Jolla, CA: GraphPad Software, Inc. Retrieved from http://www.graphpad.com/scientific-software/prism/.

[26] Casey, M., Gennings, C., Carter, W. H., Moser, V. C. & Simmons, J. E. Detecting interaction (s) and assessing the impact of component subsets in a chemical mixture using fixed-ratio mixture ray designs. *J. Agric. Biol., Environ. Stat.* **9**, 339–361 (2004).

[27] Gennings, C. *et al.* Analysis of functional effects of a mixture of five pesticides using a ray design. *Environ. Toxicol. Pharmacol.* **18**(2), 115–125 (2004). 21782740 10.1016/j.etap.2004.03.012

[28] Cedergreen, N. Quantifying synergy: a systematic review of mixture toxicity studies within environmental toxicology. *PloS one* **9**(5), e96580 (2014). 24794244 10.1371/journal.pone.0096580 PMC4008607

[29] Froimowitz, M. HyperChem: a software package for computational chemistry and molecular modeling. *Biotechniques* **14**(6), 1010–1013 (1993). 8333944

[30] Frisch, M. J. *et al.**Gaussian 09, rev* (Gaussian Inc, 2009).

[31] Del Bene, J. E., Person, W. B. & Szczepaniak, K. Properties of hydrogen-bonded complexes obtained from the B3LYP functional with 6–31G (d, p) and 6–31+ G (d, p) basis sets: Comparison with MP2/6-31+ G (d, p) results and experimental data. *J. Phys. Chem.* **99**(27), 10705–10707 (1995).

[32] Todeschini, R., Consonni, V., & Pavan, M., DRAGON–Software for the calculation of molecular descriptors, rel. 1.12 for Windows. Free download available at http://www.disat.unimib/chm (2001).

[33] Costa, A. S., Martins, J. P. A. & de Melo, E. B. SMILES-based 2D-QSAR and similarity search for identification of potential new scaffolds for development of SARS-CoV-2 MPRO inhibitors. *Struct. Chem.* **33**(5), 1691–1706 (2022). 35811781 10.1007/s11224-022-02008-9 PMC9257568

[34] Rosell-Hidalgo, A., Moore, A. L. & Ghafourian, T. Prediction of drug-induced mitochondrial dysfunction using succinate-cytochrome c reductase activity, QSAR Molecular docking. *Toxicology* **485**, 153412 (2023). 36584908 10.1016/j.tox.2022.153412

[35] Qin, L. T., Liu, S. S., Chen, F. & Wu, Q. S. Development of validated quantitative structure–retention relationship models for retention indices of plant essential oils. *J. Sep. Sci.* **36**(9–10), 1553–1560 (2013). 23441046 10.1002/jssc.201300069

[36] Gaudin, T., Rotureau, P. & Fayet, G. Mixture descriptors toward the development of quantitative structure–property relationship models for the flash points of organic mixtures. *Ind. Eng. Chem. Res.* **54**(25), 6596–6604 (2015).

[37] Tang, K. S., Man, K. F., Kwong, S. & He, Q. Genetic algorithms and their applications. *IEEE Signal Process. Mag.* **13**(6), 22–37 (1996).

[38] MATLAB, V., 2019. 9.7. 0 (R2019b). *The MathWorks Inc, Natick, Massachusetts*.

[39] Leonard, J. T. & Roy, K. On selection of training and test sets for the development of predictive QSAR models. *QSAR Combinat. Sci.* **25**(3), 235–251 (2006).

[40] Aiken, L.S., West, S.G. and Reno, R.R., 1991. *Multiple regression: Testing and interpreting interactions*. Sage. 212pp.

[41] Ghanei-Nasab, S., Hadizadeh, F., Foroumadi, A. & Marjani, A. A QSAR study for the prediction of inhibitory activity of coumarin derivatives for the treatment of Alzheimer’s disease. *Arab. J. Sci. Eng.* **46**(6), 5523–5531 (2021).

[42] Žuvela, P., David, J., Yang, X., Huang, D. & Wong, M. W. Non-linear quantitative structure–activity relationships modelling, mechanistic study and in-silico design of flavonoids as potent antioxidants. *Int. J. Mol. Sci.* **20**(9), 2328 (2019). 31083440 10.3390/ijms20092328 PMC6539043

[43] King, R. D., Hirst, J. D. & Sternberg, M. J. New approaches to QSAR: neural networks and machine learning. *Perspect. Drug Discov. Des.* **1**, 279–290 (1993).

[44] Mei, H., Zhou, Y., Liang, G. & Li, Z. Support vector machine applied in QSAR modelling. *Chinese Sci. Bull.* **50**, 2291–2296 (2005).

[45] Doucet, J. P., Barbault, F., Xia, H., Panaye, A. & Fan, B. Nonlinear SVM approaches to QSPR/QSAR studies and drug design. *Curr. Comput. Aided Drug Des.* **3**(4), 263–289 (2007).

[46] Liu, H. X. *et al.* Prediction of the isoelectric point of an amino acid based on GA-PLS and SVMs. *J. Chem. Inf. Comput. Sci.* **44**(1), 161–167 (2004). 14741023 10.1021/ci034173u

[47] Suter, B. W. The multilayer perceptron as an approximation to a Bayes optimal discriminant function. *IEEE Trans. Neural Netw.* **1**(4), 291 (1990). 10.1109/72.80266 18282850

[48] Gardner, M. W. & Dorling, S. R. Artificial neural networks (the multilayer perceptron)—a review of applications in the atmospheric sciences. *Atmos. Environ.* **32**(14–15), 2627–2636 (1998).

[49] Beale, M. H., Hagan, M. T. & Demuth, H. B. Neural network toolbox. *User’s Guide, MathWorks* **2**, 77–81 (2010).

[50] Airola, A., Pahikkala, T., Waegeman, W., De Baets, B. & Salakoski, T. An experimental comparison of cross-validation techniques for estimating the area under the ROC curve. *Comput. Stat. Data Anal.* **55**(4), 1828–1844 (2011).

[51] Huang, W. *et al.* Prediction of human clearance based on animal data and molecular properties. *Chem. Biol. Drug Des.* **86**(5), 990–997 (2015). 25845625 10.1111/cbdd.12567

[52] Golbraikh, A. & Tropsha, A. Beware of q2!. *J. Mol. Gr. Modell.* **20**(4), 269–276 (2002). 10.1016/s1093-3263(01)00123-1 11858635

[53] Kennedy, P. E. & Cade, B. S. Randomization tests for multiple regression. *Commun. Stat. Simul. Comput.* **25**(4), 923–936 (1996).

[54] Rücker, C., Rücker, G. & Meringer, M. Y-randomization–a useful tool in QSAR validation, or folklore. *J. Chem. Inf. Model* **47**, 2345–2357 (2007). 17880194 10.1021/ci700157b

[55] OECD. *Guidance Document on the Validation of (Quantitative) Structure-Activity Relationship [(Q)SAR] Models* (OECD, 2014). 10.1787/9789264085442-en.

[56] Roy, K., Kar, S. & Ambure, P. On a simple approach for determining applicability domain of QSAR models. *Chemomet. Intell. Lab. Syst.* **145**, 22–29 (2015).

[57] Todeschini, R., Consonni, V. and Gramatica, P., 2009. Chemometrics in QSAR. In *Comprehensive chemometrics* (Vol. 4, pp. 129–172). Elsevier.

[58] Gadaleta, D., Mangiatordi, G. F., Catto, M., Carotti, A. & Nicolotti, O. Applicability domain for QSAR models: Where theory meets reality. *Int. J. Quant. Struct. Prop. Relations. (IJQSPR)* **1**(1), 45–63 (2016).

[59] Geiger, E., Hornek-Gausterer, R. & Saçan, M. T. Single and mixture toxicity of pharmaceuticals and chlorophenols to freshwater algae Chlorella vulgaris. *Ecotoxicol. Environ. Saf.* **129**, 189–198 (2016). 27045919 10.1016/j.ecoenv.2016.03.032

[60] Zhang, Y.H., & Liu, Z., 2015. Study on the mixture toxicity of organophosphorus (OP) pesticides. *Toxic Pollutants in China: Study of Water Quality Criteria*, pp.129–140.

[61] Smola, A. J. & Schölkopf, B. A tutorial on support vector regression. *Stat. Comput.* **14**, 199–222 (2004).

[62] Moussaoui, M., Laidi, M., Hanini, S. & Hentabli, M. Artificial neural network and support vector regression applied in quantitative structure-property relationship modelling of solubility of solid solutes in supercritical CO 2. *Kemija u industriji: Časopis kemičara i kemijskih inženjera Hrvatske* **69**(11–12), 611–630 (2020).

[63] Lu, W. C. *et al.* Using support vector machine for materials design. *Adv. Manuf.* **1**, 151–159 (2013).

[64] Yao, X. *et al.* QSAR and classification study of 1, 4-dihydropyridine calcium channel antagonists based on least squares support vector machines. *Mol. Pharmaceut.* **2**(5), 348–356 (2005). 10.1021/mp050027v 16196487

[65] Salt, D. W., Yildiz, N., Livingstone, D. J. & Tinsley, C. J. The use of artificial neural networks in QSAR. *Pesticide Sci.* **36**(2), 161–170 (1992).

[66] Fissa, M. R., Lahiouel, Y., Khaouane, L. & Hanini, S. QSPR estimation models of normal boiling point and relative liquid density of pure hydrocarbons using MLR and MLP-ANN methods. *J. Mol. Gr. Modell.* **87**, 109–120 (2019). 10.1016/j.jmgm.2018.11.013 30537641

[67] Žuvela, P., David, J. & Wong, M. W. Interpretation of ANN-based QSAR models for prediction of antioxidant activity of flavonoids. *J. Comput. Chem.* **39**(16), 953–963 (2018). 29399831 10.1002/jcc.25168

[68] Kianpour, M., Mohammadinasab, E. & Isfahani, T. M. Prediction of oral acute toxicity of organophosphates using QSAR methods. *Curr. Comput. Aided Drug Des.* **17**(1), 38–56 (2021). 31880265 10.2174/1573409916666191227093237

[69] Hamadache, M., Benkortbi, O., Hanini, S. & Amrane, A. Application of multilayer perceptron for prediction of the rat acute toxicity of insecticides. *Energy Procedia* **139**, 37–42 (2017).

[70] Wu, Z. *et al.* Do we need different machine learning algorithms for QSAR modeling? A comprehensive assessment of 16 machine learning algorithms on 14 QSAR data sets. *Brief. Bioinform.* **22**(4), 321 (2021). 10.1093/bib/bbaa321 33313673

