# Supplementary material for: Combined interaction of fungicides binary mixtures: experimental study and machine learning-driven QSAR modeling
Source: Sci Rep. 2024 Jun 3;14:12700. doi: 10.1038/s41598-024-63708-2 (PMC11639718; doi:10.1038/s41598-024-63708-2)
Supplement: Supplementary file 1 — Supplementary Information. [file 41598_2024_63708_MOESM1_ESM.docx]

**Combined interaction of fungicides binary mixtures: experimental study and machine learning-driven QSAR modeling**

**Mohsen Abbod^1*^, Ahmad** **Mohammad^1^**

^1^ Department of Plant Protection, Faculty of Agriculture, Al-Baath University, Homs, Syria

**Corresponding author: E-mail address:** [**abbod.mohsen111@gmail.com**](mailto:abbod.mohsen111@gmail.com)

The supplementary file includes:

**Supplementary Table S1:** The calculated EC_50_ of tested mixture by CA and IA models.

**Supplementary Table S2:** The molecular descriptors chosen by GA and their values for the mixtures

**Table S1.** The calculated EC_50_ of tested mixture by CA and IA models.

| **No.** | **Mixture ray** | **EC_50, obs._**  **(mg/L)** | **EC_50, CA_** | **MDR _(CA)_** | **Interaction _(CA)_** | **EC_50, IA_** | **MDR _(IA)_** | **Interaction _(IA)_** |
| --- | --- | --- | --- | --- | --- | --- | --- | --- |
| 1 | TM-CT (R1) | 6.554 | 3.983 | 0.61 | AD | 2.730 | 0.42 | A |
| 2 | TM-CT (R2) | 4.658 | 3.774 | 0.81 | AD | 1.486 | 0.32 | A |
| 3 | TM-CT (R3) | 4.379 | 3.572 | 0.82 | AD | 0.691 | 0.16 | A |
| 4 | TM-CT (R4) | 2.798 | 3.358 | 1.20 | AD | 0.470 | 0.17 | A |
| 5 | TM-CT (R5) | 2.307 | 3.157 | 1.37 | AD | 1.184 | 0.51 | AD |
| 6 | TM-IP (R1) | 9.65 | 2.403 | 0.25 | A | 1.343 | 0.14 | A |
| 7 | TM-IP (R2) | 7.5 | 2.657 | 0.35 | A | 0.939 | 0.13 | A |
| 8 | TM-IP (R3) | 7 | 2.623 | 0.37 | A | 0.993 | 0.14 | A |
| 9 | TM-IP (R4) | 4.681 | 2.734 | 0.58 | AD | 1.402 | 0.30 | A |
| 10 | TM-IP (R5) | 4.482 | 2.847 | 0.64 | AD | 2.320 | 0.52 | AD |
| 11 | IP-CT (R1) | 2.97 | 3.865 | 1.30 | AD | 3.129 | 1.05 | AD |
| 12 | IP-CT (R2) | 1.748 | 2.549 | 1.46 | AD | 2.119 | 1.21 | AD |
| 13 | IP-CT (R3) | 1.704 | 3.248 | 1.91 | AD | 1.337 | 0.78 | AD |
| 14 | IP-CT (R4) | 1.449 | 2.930 | 2.02 | S | 0.805 | 0.56 | AD |
| 15 | IP-CT (R5) | 1.319 | 2.614 | 1.98 | AD | 0.913 | 0.69 | AD |
| 16 | DF-TM (R1) | 3.428 | 5.876 | 1.71 | AD | 4.754 | 1.39 | AD |
| 17 | DF-TM (R2) | 5.427 | 5.254 | 0.97 | AD | 2.847 | 0.52 | AD |
| 18 | DF-TM (R3) | 6.812 | 4.710 | 0.69 | AD | 1.291 | 0.19 | A |
| 19 | DF-TM (R4) | 7.197 | 4.108 | 0.57 | AD | 0.022 | 0.003 | A |
| 20 | DF-TM (R5) | 7.442 | 3.525 | 0.47 | A | 0.009 | 0.001 | A |
| 21 | DF-CT (R1) | 2.996 | 4.565 | 1.52 | AD | 0.183 | 0.06 | A |
| 22 | DF-CT (R2) | 4.137 | 4.922 | 1.19 | AD | 1.442 | 0.35 | A |
| 23 | DF-CT (R3) | 4.672 | 5.315 | 1.14 | AD | 0.844 | 0.18 | A |
| 24 | DF-CT (R4) | 5.143 | 5.668 | 1.10 | AD | 0.826 | 0.16 | A |
| 25 | DF-CT (R5) | 7.783 | 6.040 | 0.78 | AD | 3.320 | 0.43 | A |
| 26 | DF-IP (R1) | 5.554 | 2.987 | 0.54 | AD | 0.383 | 0.07 | A |
| 27 | DF-IP (R2) | 4.902 | 3.661 | 0.75 | AD | 1.097 | 0.22 | A |
| 28 | DF-IP (R3) | 2.308 | 4.379 | 1.90 | AD | 2.514 | 1.09 | AD |
| 29 | DF-IP (R4) | 2.178 | 5.062 | 2.32 | S | 4.427 | 2.03 | S |
| 30 | DF-IP (R5) | 2.076 | 5.710 | 2.75 | S | 5.179 | 2.49 | S |
| 31 | TB-CT (R1) | 3.323 | 3.525 | 1.06 | AD | 4.003 | 1.20 | AD |
| 32 | TB-CT (R2) | 2.593 | 2.915 | 1.12 | AD | 3.772 | 1.45 | AD |
| 33 | TB-CT (R3) | 2.123 | 2.396 | 1.13 | AD | 3.494 | 1.65 | AD |
| 34 | TB-CT (R4) | 1.752 | 1.767 | 1.01 | AD | 2.973 | 1.70 | AD |
| 35 | TB-CT (R5) | 0.7 | 1.179 | 1.68 | AD | 2.118 | 3.03 | S |
| 36 | TB-DF (R1) | 2.283 | 5.350 | 2.34 | S | 6.239 | 2.73 | S |
| 37 | TB-DF (R2) | 1.896 | 4.581 | 2.42 | S | 6.052 | 3.19 | S |
| 38 | TB-DF (R3) | 1.35 | 3.558 | 2.64 | S | 5.687 | 4.21 | S |
| 39 | TB-DF (R4) | 0.6773 | 2.559 | 3.78 | S | 5.076 | 7.49 | S |
| 40 | TB-DF (R5) | 0.6345 | 1.558 | 2.46 | S | 3.818 | 6.02 | S |
| 41 | TB-TM (R1) | 2.208 | 2.538 | 1.15 | AD | 2.792 | 1.26 | AD |
| 42 | TB-TM (R2) | 1.909 | 2.158 | 1.13 | AD | 2.599 | 1.36 | AD |
| 43 | TB-TM (R3) | 1.867 | 1.785 | 0.96 | AD | 2.343 | 1.26 | AD |
| 44 | TB-TM (R4) | 0.8243 | 1.376 | 1.67 | AD | 1.943 | 2.36 | S |
| 45 | TB-TM (R5) | 0.5012 | 0.970 | 1.93 | AD | 1.338 | 2.67 | S |
| 46 | TB-IP (R1) | 1.782 | 2.000 | 1.12 | AD | 2.147 | 1.20 | AD |
| 47 | TB-IP (R2) | 1.132 | 1.732 | 1.53 | AD | 1.977 | 1.75 | AD |
| 48 | TB-IP (R3) | 0.9769 | 1.442 | 1.48 | AD | 1.740 | 1.78 | AD |
| 49 | TB-IP (R4) | 0.7815 | 1.145 | 1.46 | AD | 1.577 | 2.02 | S |
| 50 | TB-IP (R5) | 0.423 | 0.864 | 2.04 | S | 1.007 | 2.38 | S |

| **No.** | **Mixture ray** | **ATS3m** | **MLOGP** | **E*_LUMO_*** | ***X*** |
| --- | --- | --- | --- | --- | --- |
| 1 | TM-CT (R1) | 61.83096 | 2.97112 | -3.0851 | 5.5131 |
| 2 | TM-CT (R2) | 58.70308 | 2.78226 | -2.8901 | 5.2762 |
| 3 | TM-CT (R3) | 55.35178 | 2.57991 | -2.6811 | 5.0223 |
| 4 | TM-CT (R4) | 51.33022 | 2.33709 | -2.4303 | 4.7177 |
| 5 | TM-CT (R5) | 47.08524 | 2.08078 | -2.1656 | 4.3962 |
| 6 | TM-IP (R1) | 47.2916 | 1.9632 | -1.8382 | 4.4057 |
| 7 | TM-IP (R2) | 46.07522 | 1.92064 | -1.8432 | 4.3151 |
| 8 | TM-IP (R3) | 44.98688 | 1.88256 | -1.8476 | 4.2339 |
| 9 | TM-IP (R4) | 43.96256 | 1.84672 | -1.8518 | 4.1575 |
| 10 | TM-IP (R5) | 43.00226 | 1.81312 | -1.8557 | 4.0859 |
| 11 | IP-CT (R1) | 62.918 | 3.0205 | -3.1104 | 5.5947 |
| 12 | IP-CT (R2) | 61.0052 | 2.8855 | -2.9401 | 5.4489 |
| 13 | IP-CT (R3) | 58.933 | 2.73925 | -2.7555 | 5.2909 |
| 14 | IP-CT (R4) | 56.2232 | 2.548 | -2.5143 | 5.0844 |
| 15 | IP-CT (R5) | 52.8758 | 2.31175 | -2.2162 | 4.8292 |
| 16 | DF-TM (R1) | 58.24332 | 2.61016 | -1.3231 | 4.0233 |
| 17 | DF-TM (R2) | 56.32151 | 2.51138 | -1.3872 | 4.0234 |
| 18 | DF-TM (R3) | 54.22499 | 2.40362 | -1.4571 | 4.0235 |
| 19 | DF-TM (R4) | 51.25492 | 2.25096 | -1.5561 | 4.0236 |
| 20 | DF-TM (R5) | 47.4113 | 2.0534 | -1.6843 | 4.0237 |
| 21 | DF-CT (R1) | 63.34296 | 3.02476 | -2.7781 | 5.3099 |
| 22 | DF-CT (R2) | 62.41747 | 2.93907 | -2.4027 | 4.9882 |
| 23 | DF-CT (R3) | 61.54069 | 2.85789 | -2.0471 | 4.6835 |
| 24 | DF-CT (R4) | 60.85875 | 2.79475 | -1.7705 | 4.4465 |
| 25 | DF-CT (R5) | 60.22552 | 2.73612 | -1.5136 | 4.2264 |
| 26 | DF-IP (R1) | 52.55684 | 2.25064 | -1.6327 | 4.3291 |
| 27 | DF-IP (R2) | 54.99202 | 2.39892 | -1.5102 | 4.2240 |
| 28 | DF-IP (R3) | 56.76306 | 2.50676 | -1.4212 | 4.1475 |
| 29 | DF-IP (R4) | 57.98065 | 2.5809 | -1.3600 | 4.0949 |
| 30 | DF-IP (R5) | 58.86617 | 2.63482 | -1.3155 | 4.0567 |
| 31 | TB-CT (R1) | 63.8355 | 3.10945 | -3.1790 | 5.6566 |
| 32 | TB-CT (R2) | 62.9335 | 3.07805 | -3.0813 | 5.5771 |
| 33 | TB-CT (R3) | 61.806 | 3.0388 | -2.9591 | 5.4778 |
| 34 | TB-CT (R4) | 59.551 | 2.9603 | -2.7148 | 5.2792 |
| 35 | TB-CT (R5) | 55.2665 | 2.81115 | -2.2507 | 4.9018 |
| 36 | TB-DF (R1) | 59.28742 | 2.67532 | -1.2672 | 4.0174 |
| 37 | TB-DF (R2) | 58.93384 | 2.66864 | -1.2578 | 4.0115 |
| 38 | TB-DF (R3) | 58.22668 | 2.65528 | -1.2391 | 3.9998 |
| 39 | TB-DF (R4) | 56.98915 | 2.6319 | -1.2064 | 3.9793 |
| 40 | TB-DF (R5) | 54.16051 | 2.57846 | -1.1317 | 3.9323 |
| 41 | TB-TM (R1) | 42.16168 | 1.80656 | -1.8171 | 4.0121 |
| 42 | TB-TM (R2) | 42.15128 | 1.83476 | -1.7646 | 3.9974 |
| 43 | TB-TM (R3) | 42.13672 | 1.87424 | -1.6911 | 3.9769 |
| 44 | TB-TM (R4) | 42.11176 | 1.94192 | -1.5652 | 3.9416 |
| 45 | TB-TM (R5) | 42.066 | 2.066 | -1.3342 | 3.8770 |
| 46 | TB-IP (R1) | 48.2415 | 2.025 | -1.7818 | 4.4626 |
| 47 | TB-IP (R2) | 47.8449 | 2.0454 | -1.7204 | 4.4164 |
| 48 | TB-IP (R3) | 47.25 | 2.076 | -1.6283 | 4.3470 |
| 49 | TB-IP (R4) | 46.3246 | 2.1236 | -1.4849 | 4.2390 |
| 50 | TB-IP (R5) | 44.8704 | 2.1984 | -1.2597 | 4.0693 |

**Table S2**. The molecular descriptors chosen by GA and their values for the mixtures.
